# Climate prediction of El Niño malaria epidemics in north-west Tanzania

**DOI:** 10.1186/1475-2875-6-162

**Published:** 2007-12-06

**Authors:** Anne E Jones, Ulrika Uddenfeldt Wort, Andrew P Morse, Ian M Hastings, Alexandre S Gagnon

**Affiliations:** 1 Department of Geography, University of Liverpool, Liverpool, UK; 2 Division of International Health (IHCAR), Department of Public Health Sciences, Karolinska Institutet, Stockholm, Sweden; 3 Liverpool School of Tropical Medicine, University of Liverpool, UK; 4 Environmental Research Institute. North Highland College, UHI Millennium Institute, Thurso, KW14 7JD, UK

## Abstract

**Background:**

Malaria is a significant public health problem in Tanzania. Approximately 16 million malaria cases are reported every year and 100,000 to 125,000 deaths occur. Although most of Tanzania is endemic to malaria, epidemics occur in the highlands, notably in Kagera, a region that was subject to widespread malaria epidemics in 1997 and 1998. This study examined the relationship between climate and malaria incidence in Kagera with the aim of determining whether seasonal forecasts may assist in predicting malaria epidemics.

**Methods:**

A regression analysis was performed on retrospective malaria and climatic data during each of the two annual malaria seasons to determine the climatic factors influencing malaria incidence. The ability of the DEMETER seasonal forecasting system in predicting the climatic anomalies associated with malaria epidemics was then assessed for each malaria season.

**Results:**

It was found that malaria incidence is positively correlated with rainfall during the first season (Oct-Mar) (R-squared = 0.73, p < 0.01). For the second season (Apr-Sep), high malaria incidence was associated with increased rainfall, but also with high maximum temperature during the first rainy season (multiple R-squared = 0.79, p < 0.01). The robustness of these statistical models was tested by excluding the two epidemic years from the regression analysis. DEMETER would have been unable to predict the heavy El Niño rains associated with the 1998 epidemic. Nevertheless, this epidemic could still have been predicted using the temperature forecasts alone. The 1997 epidemic could have been predicted from observed temperatures in the preceding season, but the consideration of the rainfall forecasts would have improved the temperature-only forecasts over the remaining years.

**Conclusion:**

These results demonstrate the potential of a seasonal forecasting system in the development of a malaria early warning system in Kagera region.

## Background

*Plasmodium falciparum *malaria is a major public health problem in Tanzania. With an estimated 16 million episodes and 100,000 to 125,000 deaths reported annually out of a population of approximately 34.5 million, malaria is the leading cause of hospital attendance and mortality in the country, affecting both children and adults [1-3]. Most of Tanzania is endemic to malaria. Nonetheless, epidemics occur in the highlands and the highland-fringe areas along the Rift Valley, where approximately 8.4 million people live, representing 25% of the country's population [4,5]. Malaria epidemics in those regions are often devastating in terms of morbidity and mortality because of low immunity among the general population [6,7]. The highlands of Africa have historically been considered free of malaria transmission. Nevertheless, these areas have experienced a progressive rise in malaria incidence over the last 50 years, largely as a consequence of changes in climate, land use, drug resistance, the cessation of malaria control activities, and development in agro-forestry [8]. In these epidemic prone regions, a sharp, but temporary, increase in disease incidence occurs when the equilibrium between humans, parasites, and the mosquito vector population is disturbed [9]. Malaria epidemics in the highlands are often associated with climatic conditions favourable to the mosquito part of its life cycle (see below), but the extent of an epidemic also depends on the patient's nutritional status [10,11], and the presence of co-infections, notably HIV [12].

Previous research has identified a link between climatic anomalies and malaria incidence in a number of countries [13-15]. Rainfall is largely responsible for creating the conditions that allow sufficient surface water for mosquito breeding sites and is therefore recognized as one of the major factors influencing malaria transmission [9]. Temperature also plays a role in malaria transmission through its influence on the development rate of mosquito larvae and the survival rate of adult mosquitoes [7]. Moreover, at warmer temperatures, adult female mosquitoes feed more frequently and digest blood more rapidly and the *Plasmodium *parasite matures more rapidly within the female mosquitoes [16,17]. Further, a relative humidity of at least 60% is often regarded as a requirement for malaria transmission [18].

Few studies have been undertaken on the association between climatic conditions and malaria epidemics in Tanzania and other East African countries. Lindblade *et al *associated a malaria epidemic in a highland region of SW Uganda to unusually heavy rains engendered by the very strong 1997–1998 El Niño [19]. Similarly, Mouchet *et al *linked the 1994 malaria epidemic in the same region to high rainfall during the preceding two months [20]. Conversely, Lindsay *et al*, who compared the level of malaria infection in children before and following the 1997–1998 El Niño in the Usambara mountains of NE Tanzania, found that even though El Niño led to more abundant rainfall, fewer malaria cases were reported following this event than in the previous year, suggesting that heavy rainfall may have washed away mosquito breeding sites [21]. Also in NE Tanzania, Matola *et al *found that warmer temperatures were correlated with increased malaria incidence [22]. A similar link between warmer temperatures and malaria transmission was observed in neighbouring Rwanda [23]. Furthermore, Bonora *et al *[24] blamed higher temperatures in the last few years for the expansion of malaria to higher altitudes in the highlands of East Africa [24], although there is controversy on this association [25-27]. Hay *et al *did not find a significant change in the climate at four high-altitude locations in East Africa where malaria has been increasing since 1976 [25]. Patz *et al *[26] contended the validity of those results by mentioning that Hay *et al *[25] had made inappropriate use of a global climate dataset. These results were further contested by Zhou *et al*, who linked variability in malaria cases in seven highland sites in Ethiopia, Kenya and Uganda to synergistic effects of temperature and rainfall, and attributed the re-emergence of highland malaria to increased climate variability [27].

Malaria Early Warning Systems (MEWS) based on climate variations have been proposed to warn ministries of health of the potential of increased risk of malaria epidemics [28]. A document providing a framework for setting up a malaria early warning system has been published by the 'Roll-Back Malaria' programme of the WHO [29]. The monitoring of rainfall forms the basis component of a MEWS [30]. Hence the International Research Institute for Climate Prediction (IRI) has developed an online resource freely available to national malaria control programmes that produces maps of epidemic malaria risk based on rainfall anomalies in sub-Saharan Africa [31]. The situation is more complex, however, in the highlands of East Africa where temperature must also be taken into account. Githeko *et al *have developed a malaria prediction model for the highlands of western Kenya based on empirical studies on the association between rainfall, unusually high maximum temperatures and the number of malaria cases [32]. Seasonal climate forecasts could be used in a MEWS to extend the epidemic warning time by several months. Such a technique is currently being used in southern Africa where seasonal climate forecasts are converted to seasonal malaria forecasts by the Southern Africa Malaria Control (SAMC) authority [28].

The El Niño Southern Oscillation (ENSO) refers to an irregular cycle of warming and cooling of the sea surface temperatures (SSTs) in the eastern equatorial Pacific Ocean. This oceanic warming and cooling named El Niño and La Niña, respectively, is accompanied by changes in air pressure patterns above the Pacific Ocean and affects not only the local atmospheric circulation but also has repercussions on the climate worldwide through 'teleconnections' in the atmosphere [33,34]. Major El Niño events occur at irregular intervals varying from two to seven years and are associated with above average rainfall in East Africa [35,36]. Seasonal forecasting is based on the impact of slowly changing surface conditions such as SSTs on the atmosphere. Thus advances made in the prediction of El Niño in the past decade have improved our ability to forecast temperature and rainfall conditions up to a few months in advance [37,38].

Seasonal forecasts of temperature and rainfall have the potential to provide important societal benefits, particularly for the prediction, control, and prevention of climate-sensitive diseases such as malaria. The incorporation of seasonal forecasts in MEWS has previously been proposed [39,40]. The possibility of predicting malaria epidemics with the help of climate information offers the opportunity for intensifying control measures at times of greater risk in order to reduce morbidity and mortality. Prerequisites for the use of integrated seasonal forecasts – MEWS in a particular region include sufficient forecasting skill and that a link between climate variability and malaria incidence is established through empirical research. Thus, an understanding of the local factors affecting malaria transmission is crucial for the development of climate-based epidemic predictions.

**Figure 1:**
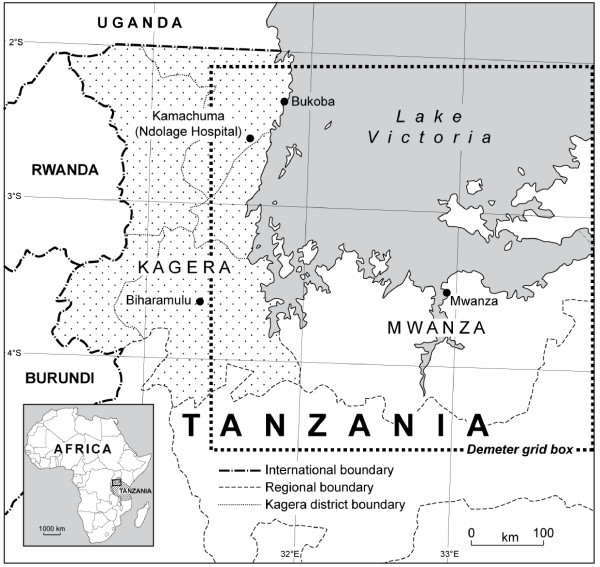
Map of the study area.

The Kagera region is situated in the highlands of NW Tanzania (Figure 1). Kagera is the most remote and one of the poorest regions of Tanzania (Bureau of Statistics, Ministry of Finance, unpublished data). The administrative centre of the region is Bukoba, located on the shore of Lake Victoria. Although the Usambara Mountains and central Tanzania are normally considered the areas with the greatest risk of malaria epidemics in the country, the NW is also prone to malaria epidemics. In fact, a widespread malaria epidemic affected Kagera region from October 1997 to May 1998 when 133,866 cases and 1,740 deaths were reported [41]. The objectives of this paper are (1) to determine the climatic conditions associated with malaria epidemics in NW Tanzania, and (2) to determine the ability and the limitations of a seasonal climate forecasting system in predicting malaria epidemics in this region.

## Methods

### Data

Retrospective data on peripheral blood smear examinations were collected from January 1990 to December 1999 from the laboratory archives of Ndolage hospital in Kagera region. This 200-bed hospital, situated 60 km south of Bukoba at an altitude of 1,600 m, is administered by the Evangelical Lutheran Church of Tanzania. The hospital routinely takes blood smears from all in and out-patients with malaria-like symptoms, thereby confirming the malaria cases microscopically [42]. Malaria is the main cause of mortality at this hospital and an increase in the incidence of this disease has been reported over the last decade [43]. Such an increase in malaria transmission has been reported in many sub-Saharan countries partly because of resistance to antimalarial drugs [44]. As a consequence of the development of resistance to chloroquine, suphadoxine/pyrethamine became the preferred drug in August 2001 in Tanzania [45]. Nonetheless, this change in clinical practice should not affect our results since it has occurred following the end of our study period.

Daily data on rain, humidity, and minimum and maximum temperature were obtained from Bukoba, the closest meteorological station to Ndolage hospital, for the same time period. These climatic data were aggregated into monthly values for comparison with the epidemiological data. In order to determine the predictability of the anomalous climatic conditions associated with malaria epidemics in NW Tanzania, we extracted the seasonal re-forecasts produced by the EU DEMETER (Development of a European Multi-model Ensembles Forecast System for Seasonal to Interannual Climate Prediction) project from the Meteorological Archival and Retrieval System [46], the main depository of meteorological data at the European Centre for Medium-Range Weather Forecasting (ECMWF). Daily DEMETER data can be found on the ECMWF public data server [47]. These seasonal re-forecasts refer to forecasts for past time-periods, so their accuracy was assessed by comparing them to the actual conditions that occurred during the forecasting period. The DEMETER seasonal forecasts of temperature and precipitation were obtained for a 2.5 degree resolution grid (approximately 250 km square) point centred on 2.5°S, 32.5°E and thus covering Kagera region (Figure 1).

The DEMETER system incorporates seven global atmosphere-ocean general circulation models and each model was integrated forward in time using nine different sets of initial conditions, therefore producing an ensemble of 63 (9 × 7) forecasts for each starting date. For this reason, DEMETER does not only predict the most likely evolution of the climate but also the uncertainty associated with it. This uncertainty arises from errors in the model formulation and in the initial conditions, as the atmosphere is an inherently chaotic system and is thereby sensitive to the initial conditions used to run the models. DEMETER produces six-month forecasts four times per year, starting on the first of February, May, August, and November. A complete description of the DEMETER project can be found in an article by Palmer *et al *[46] and on the ECMWF webpage [47].

**Figure 2:**
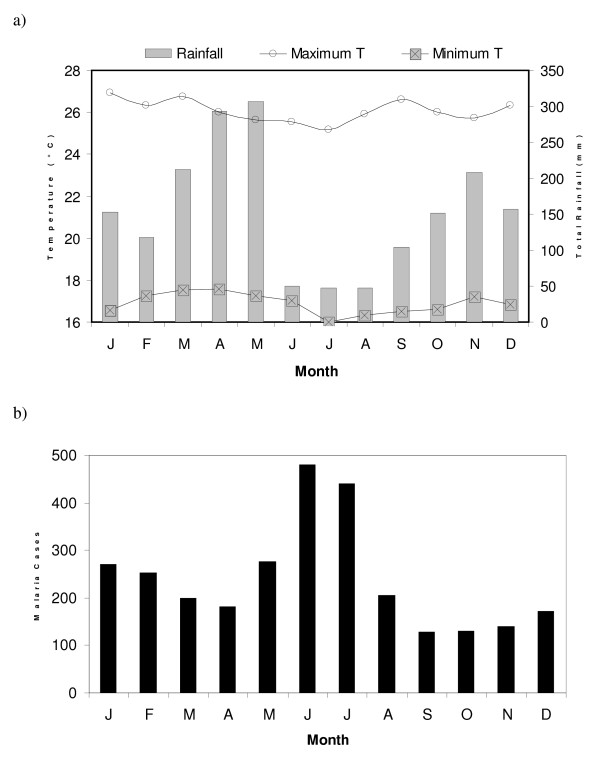
Mean monthly climatic data at Bukoba (a) and malaria cases reported by Ndolage hospital (b) for the period 1991–1999.

NW Tanzania experiences an equatorial climate with two annual rainy seasons. The short rains normally occur between October and December and the long rains between March and May (Figure 2a) [48,49]. However, for the purpose of this study, the two rainy seasons were defined by the two six-month periods extending from August to January (first season) and February to July (second season). The transmission of malaria in Kagera region follows this seasonal cycle with peaks in malaria incidence occurring one to two months following the month of highest rainfall (Figure 2b). The lowest malaria transmission occurs in September, thus the following month was defined as the start of the malaria year, with nine years occurring during the data period. The two malaria seasons corresponding to the above two rainy seasons were defined by the October to March (first season) and April to September (second season) six-month periods. This estimate of the time delay between rainfall and malaria incidence is consistent with previous studies in neighbouring Uganda [50] and other East African highland regions[27]. This time lag is also biologically plausible, as time is needed for the vector's population to increase following the emergence of newly established breeding sites and for the life cycles of the *Plasmodium *parasites to be completed in both the mosquito and the human host [51].

### Statistical analyses

A malaria index was developed for each malaria season using the number of malaria cases reported by Ndolage hospital (i.e., the total number of slides positive to malaria) during the period 1991 to 1999. This index was created by calculating the logarithm of the malaria cases and then the time series was normalized. The same method was applied to climatic data from Bukoba station to create normalized time series of variation in seasonal temperature (maximum and minimum), humidity, and total rainfall. The relationship between variation in climatic variables and malaria incidence was subsequently examined through multiple linear regression analysis for each rainy season using the R software (ve 2.3.1). This dataset is small in statistical terms (a maximum of nine observations in any one season) so it is not possible to simply include all explanatory variables in a single analysis. The putative predictor variables were therefore initially investigated singly to find those with the greatest predictive power. Subsequent analysis did include combinations of two predictors which had individually been shown to have a significant effect, the limitations of which are discussed later. To further clarify the expected dependency of malaria on rainfall and temperature, the Mapping Malaria Risk in Africa model [52] was applied to the 1991–1999 monthly averages of mean temperature and total rainfall at Bukoba station. This fuzzy logic model converts climatic data to a climate suitability index to malaria transmission ranging from zero to one; a value of one predicting that the climatic conditions are suitable to malaria transmission and a value of zero predicting unsuitable conditions.

Next, the capacity of the DEMETER forecasting system in predicting the climatic variables associated with malaria epidemics in Kagera region was assessed. Considering the timing of the two rainy seasons, only the six-month integration of the DEMETER re-forecasts started in August and February were analysed. For each ensemble member, the seasonal forecasts of temperature and rainfall were expressed as departures from the average values for the study period, thereby removing the need for bias-correction of the forecasts. A bias arises in the model forecast, because numerical models often drift away from the real climate and create their own model climate, which can be significantly different from the observed one. The calculation of the forecasts' anomalies eliminates the models' bias and ensures that the anomalies predicted by each of the 63 DEMETER ensemble members are comparable to those observed. Furthermore, the retrospective DEMETER forecasts of temperature and rainfall were combined together using the regression models developed in the first part of this study to produce malaria forecasts. These malaria forecasts were then compared to the actual peak years in malaria incidence.

## Results

### Climate – malaria relationship

**Figure 3:**
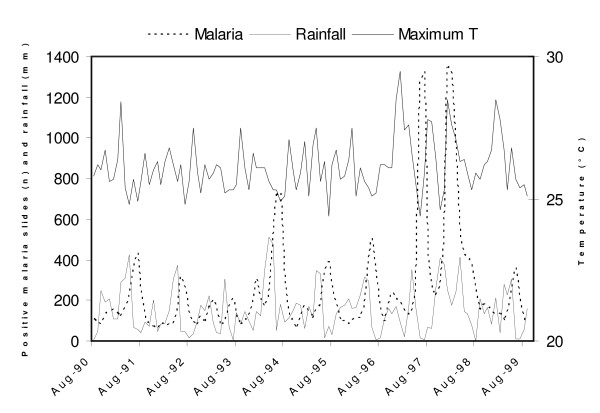
Time series of malaria, rainfall, and maximum temperature at Bukoba.

**Figure 4:**
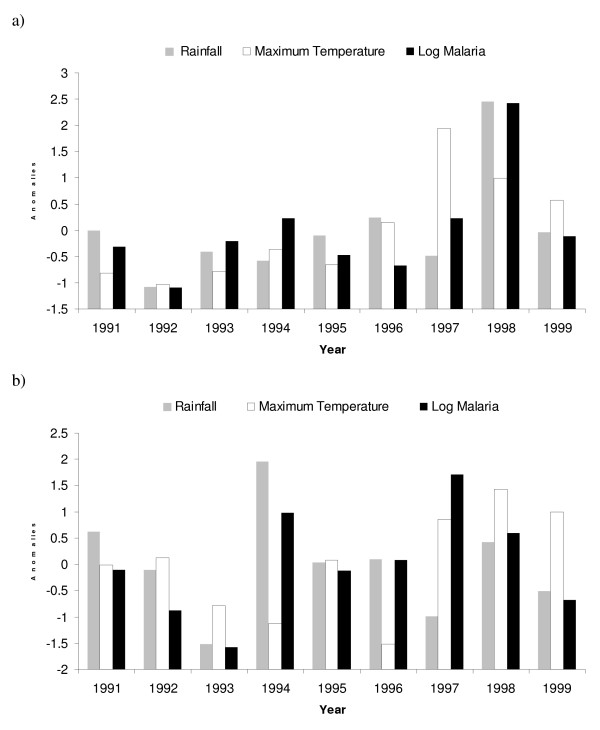
**Anomalies in climatic variables (rainfall and maximum temperature) and log malaria for the period 1991–1999 for the first (a) and second (b) season**. The first season refers to the climatic anomalies during the August to January period and the log malaria anomalies from October to March, while the second season corresponds to the climatic anomalies during the February to July period and the log malaria anomalies from April to September. The anomalies were standardized by subtracting the mean and dividing by the standard deviation.

The relationship between climatic variables and malaria incidence was first examined for the entire nine-year dataset (Figure 3). The results of a linear regression analysis reveal that the logarithm of the malaria cases during the first season (Oct-Mar) is strongly influenced by total rainfall during the Aug-Jan period (model 1; Table 1). A weak correlation but lacking statistical significance was also observed between log malaria and the average maximum temperature during the above rainy season (model 2). The correlation between log malaria and minimum temperature was much weaker (R-squared = 0.21, p = 0.25) and no correlation was found with humidity. The time series of the normalized log malaria and total rainfall and maximum temperatures are displayed in Figure 4a. The robustness of this statistical model is further supported by the calculation of the adjusted R-squared, which takes into consideration the number of degrees of freedom [53] (Table 1).

**Table 1:** Results of various multiple linear regression analyses of temperature and rainfall on log malaria incidence in Kagera region^a^.

				*Climate season*								
				Season 1 (Aug-Jan)		Season 2 (Feb-Jul)						
Malaria season	Epidemic year	n	model	Total rainfall	Max. temp.	Total rainfall	Max. temp.	BIC score	R-squared	Adjusted R-squared	Overall p-value	Comments
Season 1 (Oct-Mar)	1998	9	1	p = 0.003	n/a	n/a	n/a	19.17	0.73	0.70	p = 0.003	
Season 1 (Oct-Mar)	1998	9	2	n/a	p = 0.12	n/a	n/a	27.75	0.31	0.21	p = 0.12	
Season 1 (Oct-Mar)	1998	8	A1	p = 0.71	n/a	n/a	n/a	15.24	0.02	-0.15	p = 0.77	Excluding epidemic year 1998
Season 1 (Oct-Mar)	1998	8	A2	n/a	p = 0.14	n/a	n/a	12.27	0.32	0.21	p = 0.14	Excluding epidemic year 1998
Season 2 (Apr-Sep)	1997	9	3	n/a	n/a	p = 0.28	n/a	29.44	0.17	0.05	p = 0.28	
Season 2 (Apr-Sep)	1997	9	4	n/a	p = 0.04	n/a	n/a	25.36	0.47	0.39	p = 0.04	
Season 2 (Apr-Sep)	1997	9	5	n/a	p = 0.005	p = 0.02	n/a	19.08	0.79	0.72	p = 0.009	
Season 2 (Apr-Sep)	1997	8	B1	n/a	n/a	p = 0.002	n/a	14.34	0.82	0.79	p = 0.002	Excluding epidemic year 1997
Season 2 (Apr-Sep)	1997	8	B2	n/a	n/a	n/a	p = 0.96	28.22	0	-0.17	p = 0.96	Excluding epidemic year 1997
Season 2 (Apr-Sep)	1997	8	B3	n/a	p = 0.31	n/a	n/a	26.74	0.17	0.03	p = 0.31	Excluding epidemic year 1997
Season 2 (Apr-Sep)	1997	8	B4	n/a	p = 0.03	p = 0.001	n/a	8.4	0.94	0.91	p = 0.001	Excluding epidemic year 1997
Season 2 (Apr-Sep)	1997	6	C1	n/a	n/a	p = 0.005	n/a	9.42	0.89	0.86	p = 0.005	Excluding 1997/8/9
Season 2 (Apr-Sep)	1997	6	C2	n/a	n/a	n/a	p = 0.54	21.9	0.10	-0.13	p = 0.54	Excluding 1997/8/9
Season 2 (Apr-Sep)	1997	6	C3	n/a	p = 0.26	n/a	n/a	20.35	0.30	0.13	p = 0.26	Excluding 1997/8/9
Season 2 (Apr-Sep)	1997	6	C4	n/a	p = 0.07	p = 0.004	n/a	3.86	0.97	0.95	p = 0.005	Excluding 1997/8/9

^a ^Displayed in the table are the significance levels of each parameter included in the regression model. Each of models 1 to 5 is based on the 1991–1999 period. Models A1 and A2 refer to the first malaria season and were developed by excluding the 1998 epidemic year. The B models refer to the second malaria season and were developed by excluding the 1997 epidemic year. The C models are the same as their B equivalent but were based on the 1991–1996 period only. (n/a) Not calculated

For the second malaria season (Apr-Sep), log malaria was found to be weakly and insignificantly correlated with total rainfall during the corresponding rainy season (model 3; Table 1). No correlation was identified with maximum temperature, minimum temperature, or humidity (Figure 4b). However, a statistically significant correlation was detected between log malaria and the average maximum temperature from the first season (model 4). Furthermore, the best model fit was obtained using rainfall from the second season and maximum temperature from the first season based on both the Akaike and the Schwarz's Information Criteria (AIC, BIC, respectively; model 5). It is problematic simultaneously fitting two variables to such a small data set (most commentators recommend at least 10 observations per variable) because the results may not be robust. This was addressed in two ways. Firstly, the results for each variable are presented individually. Additional variables are then added to the one having the greatest predicting power based on the results of the AIC and BIC. Secondly, the linear models were subject to regression diagnostic tests.

An examination of the Cook's distance plots reveals that the 1997 and 1998 epidemic years influenced significantly the fit of both models. As a way to test the robustness of the above regression models, the same analysis was repeated by excluding those two epidemic years, i.e., the years 1997 and 1998 were removed from the regression analysis on the second and first malaria season, respectively. The regression of rainfall on the remaining eight years of malaria data indicates that rainfall no longer influences log malaria incidence during the first season (model A1; Table 1). A weak correlation between log malaria and maximum temperature is still observed when the 1998 epidemic year is excluded from the regression analysis (model A2). No correlation was found between log malaria and minimum temperature, but, in contrast to the analysis based on nine years of data, a weak correlation was detected between log malaria and humidity with the eight-year dataset (R-squared = 0.40, p = 0.094). Even though the addition of humidity to the linear model of maximum temperature on log malaria incidence increased the explanatory value of the model, the model still failed to reach statistical significance (R-squared = 0.50, p = 0.173) and the value of the BIC was not significantly different from the regression of maximum temperature alone on log malaria.

For the second malaria season extending from April to September, a statistically significant correlation is still observed between log malaria and total precipitation during the corresponding rainy season after removal of the 1997 epidemic year (model B1; Table 1). Moreover, no correlation is observed between log malaria and maximum temperature during the February to July rainy season (model B2). Nevertheless, as was the case with the nine-year dataset, the best model fit is obtained with total rainfall during the February-July period and maximum temperature during the preceding rainy season (model B3):

*Predicted log malaria = 0.88 [0.69 to 0.89]R + 0.461 [0.10 to 0.89] T + 0.00 [-0.23 to 0.43]*,

where R and T refer to the anomalies in total precipitation and maximum temperature, respectively. Also displayed in the equation are the 95% bootstrap confidence intervals for the regression parameters. The effect of maximum temperature during the first rainy season was not significant on its own (model B4), and only became significant when rainfall during the February-July period was included. Presumably this was because rainfall removed a substantial portion of the variation in the number of malaria cases making it easier to detect the additional effect of temperature. However it is important to note the small sample size in the analysis (8 seasons) and that regression may became instable at small sample sizes. Instability does not appear to be responsible for the result, because the magnitude of the individual regression coefficients did not change markedly when they were fitted simultaneously and significance occurred as a consequence of much smaller standard errors. Nevertheless the results need to be interpreted with some caution.

**Figure 5:**
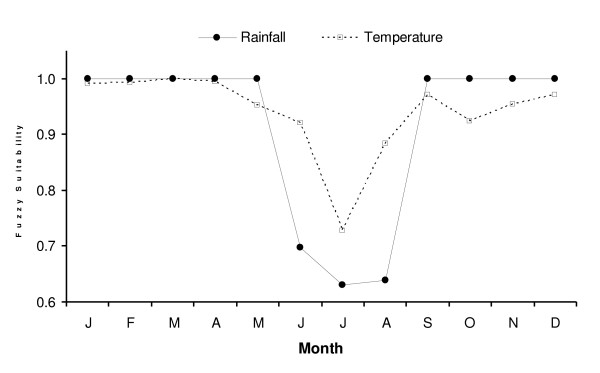
**Climatic suitability to malaria transmission according to the MARA fuzzy suitability model based on the climate data (mean temperature and total precipitation) from Bukoba station**. Mean temperature was calculated as the average of maximum and minimum temperature. The variable with the lowest suitability value is the limiting variable for malaria transmission during this particular month.

In addition, the regression of these two climatic variables on log malaria for the six-year period preceding the epidemic year of 1997 provides further evidence of the robustness of the regression model, as a statistically significant linear model is still observed (model C3). A weak correlation was also found between humidity and log malaria (R-squared = 0.38, p = 0.10). However, the BIC reveals that the best model fit is obtained with rainfall from the second season and maximum temperature from the first season. These results are further supported by outputs of the MARA fuzzy model, which indicate that a combination of rainfall and temperature limit the suitability of Kagera region to malaria transmission during the dry winter months (Figure 5).

### Seasonal climate forecasts

**Figure 6:**
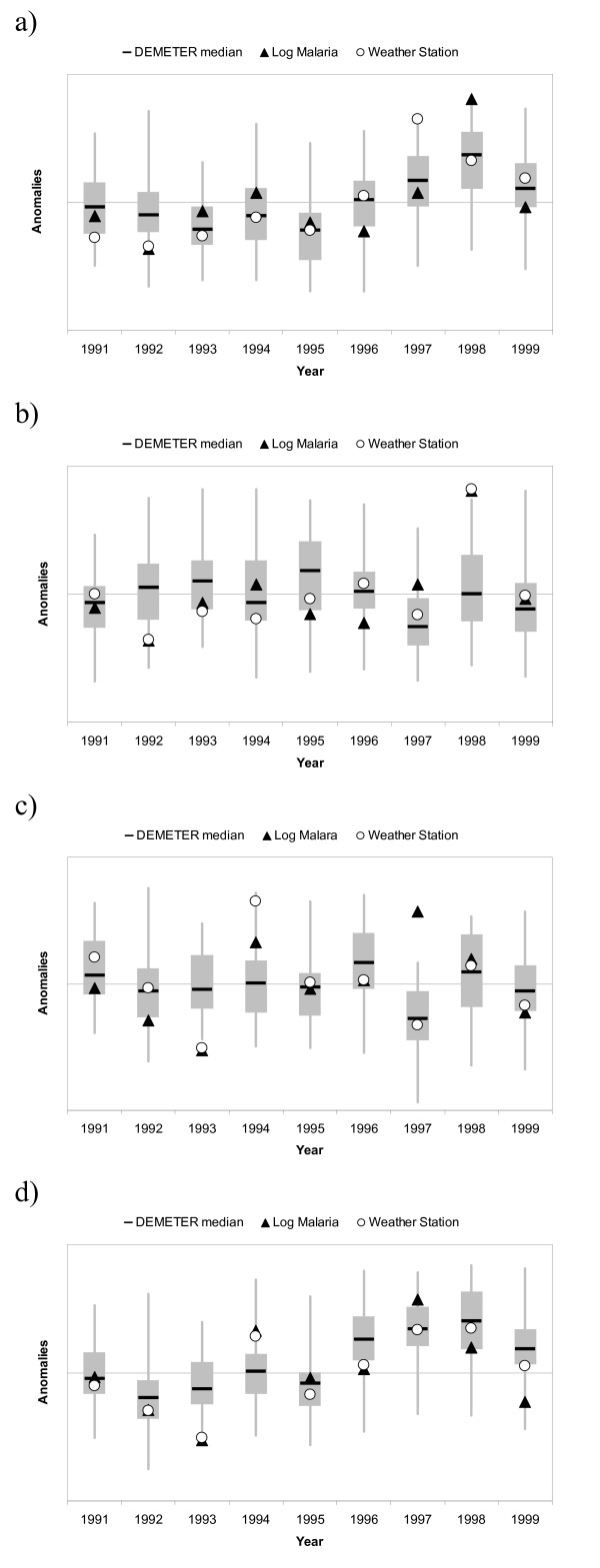
**Box plots of the anomalies of the DEMETER re-forecasts over Kagera region. Also plotted are the observed climatic anomalies at Bukoba station and the anomalies in log malaria incidence data reported at Ndolage hospital**. The horizontal line shows the median while the edges of the box represent the upper and lower quartiles of the 63 DEMETER ensembles. The ends of the whiskers show the minimum and maximum values of the DEMETER re-forecasts. First, the DEMETER forecasts of maximum temperature anomalies for the Aug-Jan season are compared to the observed temperature and malaria anomalies (a). Second, the DEMETER forecasts of total rainfall during the same period are compared to the corresponding observed rainfall and malaria anomalies (b). Third, the DEMETER forecasts of total rainfall during the second rainy season (February starting date) are compared to the corresponding observed rainfall and malaria anomalies (c). Fourth, the six-month DEMETER forecasts of total rainfall during the same period are combined with the observed maximum temperature anomalies during the first rainy season using the 'C3' linear regression model. These resulting malaria forecasts anomalies are compared to the weather observation predicted malaria and observed malaria anomalies (d). The anomalies were standardized by subtracting the mean and dividing by the standard deviation.

The potential for forecasting the anomalous climatic conditions that were previously found to be associated with years of high malaria incidence was assessed using the DEMETER multi-model ensemble forecasting system. Firstly, the anomalies in maximum temperature predicted by all 63 DEMETER ensemble members during the first rainy season (Aug-Jan) were compared to the observed anomalies in maximum temperature at Bukoba as well as with the log malaria anomalies during the October to March period. Given the short duration of the dataset, skill scores could not be used to quantify the skill of those forecasts, but the spread among the ensemble members provides a good indication of the confidence in those forecasts. Figure 6a shows that the maximum temperature anomalies from the DEMETER models agree reasonably well with the observed temperature anomalies at Bukoba with the exceptions of 1992 and 1997 when the station anomalies fell outside the inter-quartile range of the DEMETER ensemble members. The regression analysis of rainfall and temperature on log malaria for the first season revealed that the 1998 epidemic was mainly the result of anomalous rainfall conditions, whereas the variability in malaria over the remaining years was weakly correlated with maximum temperature. All DEMETER ensemble members falling within the inter-quartile range correctly predicted above average maximum temperature during the 1998 epidemic. For five out of the eight remaining years the observed malaria anomalies fell within the inter-quartile of the DEMETER forecast temperature anomalies. In 1992, both observed malaria and station temperature were below average whereas only the median of the forecast anomalies was negative. In 1996, however, DEMETER predicted average conditions and even though average maximum temperature was observed at Bukoba, the incidence of malaria was below average during this year. In 1999, the forecast and observed temperature anomalies were positive but near normal malaria conditions were recorded at the hospital.

Secondly, the anomalies in total rainfall predicted by all DEMETER models during the first season (Aug-Jan) were compared to the observed anomalies in total rainfall at Bukoba as well as with the log malaria anomalies for the corresponding malaria season. As expected, Figure 6b illustrates the greater uncertainty involved in forecasting rainfall than temperature. For example, the DEMETER system was unable to predict the anomalous rainfall conditions that were associated with the 1998 epidemic. Although some DEMETER ensemble members predicted above average rainfall during the Oct-Mar period, as many forecasted below average rainfall so that the median of the DEMETER forecasts was near zero.

Thirdly, the anomalies in total rainfall predicted by DEMETER for the second season (Feb-Jul) were compared to the observed rainfall anomalies at Bukoba and the log malaria anomalies for the corresponding malaria season (Apr-Sep). Figure 6c shows a better agreement between the DEMETER rainfall forecasts and the observed rainfall anomalies at Bukoba for the second season than the first season, as the anomalies at the weather station lie within the inter-quartile range of the 63 DEMETER forecasts for seven of the nine years. However, DEMETER failed to predict the anomalously low rainfall anomalies of 1993 as well as the high rainfall anomalies of 1994, which were associated with high malaria incidence. Nevertheless, the DEMETER rainfall forecasts for the 1997 epidemic year were close to the observed rainfall, both consisting of below average anomalies.

Fourthly, the "C3" multiple linear regression model was used to combine total rainfall during the second rainy season with the average maximum temperature from the first rainy season to predict malaria incidence for the April-September season. This provides an example of the reliability and uncertainty that would have been involved had this forecasting model been used operationally back in 1997. In reality the B3 model would be preferred since it involves a larger number of observations and should thus be more robust but, back in 1997, the data for the years 1998 and 1999 were not available (the C3 model consists of the same variables as the B3 model, but it was built using data until 1996 only). Figure 6d shows a box plot of the combination of the DEMETER forecasts of rainfall anomalies started in February combined with the anomalies of maximum temperature observed during the previous rainy season. Also displayed on the figure are the observed anomalies in log malaria and those predicted by the C3 regression model using climatic data from Bukoba station. As expected, a strong relationship is observed between the observed malaria anomalies and those predicted using the regression model. An exception is 1999 when near normal conditions would be expected by applying the regression model to the station data, but below average malaria anomalies were observed. The DEMETER forecasts correctly predicted 1997 and 1998 as anomalous years in malaria incidence. In 1994, however, the DEMETER-driven model predicted normal conditions and not the relatively high malaria incidence that was observed. This is consistent with DEMETER failing to forecast the 1994 rainfall anomaly as shown in Figure 6c. Moreover, if DEMETER had been used operationally, the seasonal forecast for 1996 would have resulted in a false alarm, as the wetter conditions predicted by DEMETER (Figure 5c) were not observed at Bukoba and, hence, the incidence of malaria was normal.

## Discussion

This paper examined malaria incidence and climatic data for Kagera region in NW Tanzania in an attempt to identify the climatic factors correlating with high malaria transmission years. A multiple linear regression analysis on the nine-year dataset shows that for the first season (Oct-Mar), the log malaria cases were strongly and positively correlated with rainfall during the corresponding rainy season. For the second season (Apr-Sep), log malaria was found to be associated with rainfall during the corresponding rainy season, but also with maximum temperature during the first season. The analysis was repeated with the exclusion of the 1997 and 1998 epidemic years. A weak and positive correlation between log malaria and maximum temperature was found for the first season, but the results for the second season were not affected by the exclusion of the 1997 epidemic year.

The anomaly plots for the first season and the correlation results for the whole nine-year period confirm that the 1998 epidemic was associated with excessive rainfall along with above average maximum temperatures. Cox *et al *also associated the 1998 epidemic to the unusually heavy El Niño-related rains, although they noted that the malaria situation was aggravated by the lack of anti-malarial drugs and resistance to chloroquine [41]. Based on data excluding this epidemic, there would be no statistical basis for assuming a link between excessive rainfall and high malaria incidence during the first season, as the correlation results suggest that malaria variability is associated with maximum temperature only. It may be the case that there is no sensitivity to rainfall under "normal" conditions, but there is a threshold above which excessive rainfall can trigger epidemics. Although the multiple regression analysis has revealed that malaria transmission in the second season is influenced by precipitation during the corresponding rainy season and maximum temperatures from the previous season, the 1997 epidemic was not preceded by heavy rainfall.

In reality, the underlying relationship between rainfall and malaria in this location may be too complicated to be revealed by this type of analysis. Other studies have found a complex and non-linear relationship between rainfall and malaria [21,54], citing explanations such as an increase in stable mosquito breeding sites in low rainfall conditions and washing out of breeding sites in heavy rain. Nevertheless, maximum temperature was above average during the first rainy season. Temperature has an exponential effect on parasite development so even a small increase in temperature can accelerate significantly the development of the parasite [55]. Moreover, the rate of larval development and hence the subsequent increase in the size of the mosquito population is also dependent on water temperature [56]. In Kagera region, environmental temperatures are relatively high year-round, but are lowest during the second season when mean monthly maximum temperatures fall below 26°C (Figure 2). Even though the MARA fuzzy suitability model suggests that temperature would decrease the suitability of the region to malaria transmission during the second rainy season (Figure 4), malaria incidence from April to September was found to be correlated with maximum temperatures during the first rainy season and not with temperatures during those cooler months.

The cause of the observed relationship between temperature anomalies in the first rainy season and malaria incidence in the following season is not clear. Nevertheless, other studies have identified a similar lag time between temperature and high malaria incidence in other African countries. Freeman and Bradley noted that in Zimbabwe, warmer temperatures in September were linked to an increase in the severity of malaria in the following year [57]. Zhou *et al *also observed that maximum and minimum temperatures were significantly correlated with malaria transmission two to five months later at study sites in Ethiopia, Kenya and Uganda [27]. Moreover, Craig *et al *found that the two most significant variables to malaria transmission in KwaZulu-Natal, South Africa, were mean maximum daily temperature during the preceding January to October period and total rainfall during the current summer months (Nov-Mar) [58]. They suggested that temperature during the preceding rainy season might determine the size of the reservoir of parasites or the survival rate of mosquitoes, consequently making an increase in malaria cases more likely following the onset of the rains.

A key operational question that arises is whether it would have been possible to predict the 1997 and 1998 epidemics using the DEMETER seasonal forecasts of rainfall and temperature. These results indicate that both epidemic years would have been predicted with a lead time of at least four months had DEMETER been used operationally. The 1998 epidemic occurred during the first malaria season, whereas the 1997 epidemic took place during the second season. Despite the strong association between the 1998 epidemic and excessive rainfall, the use of the DEMETER temperature forecasts alone would have correctly predicted this epidemic. However, it would also have classified 1999 as a year with a relatively high malaria incidence, when in reality near normal malaria conditions were reported during that year. However a rise in immunity is likely to have followed the 1997 and 1998 epidemics, thereby reducing the vulnerability of the local population to another epidemic, which may explain the low incidence for 1999. By combining the DEMETER forecasts of rainfall for the second season with the average maximum temperature during the first season, the 1997 epidemic would also have been correctly forecast. Since the statistical model uses temperature from the previous Aug-Jan period to predict malaria incidence during the Apr-Sep period, observed temperature alone could potentially give a warning of a forthcoming epidemic with at least a three-month lead-time. Nevertheless, the use of the DEMETER rainfall forecasts from February in combination with the observed temperature anomalies from the previous rainy season would increase the skilfulness as well as the lead-time of the malaria early warning system.

In East Africa, including Kagera region, El Niño is associated with an enhancement of rainfall during the 'short' rains (Oct-Dec), but it has little influence on the 'long' rains, which occur a few months later [36], El Niño has been shown to be predictable on seasonal timescales (Goddard *et al*. 2001), and both statistical models based on SSTs and dynamical models have had some success in predicting the short rains in East Africa [59,60]. These results indicate that the DEMETER system, however, was not able to forecast the 'short' season rainfall anomaly of 1998, although we have only considered a single DEMETER grid point and verification against a single rain gauge, therefore this may not be an universal result for East Africa. The DEMETER rainfall forecasts were more closely associated with the observed rainfall conditions during the second season, despite the lack of a link with El Niño, although the most extreme anomalies were not successfully predicted.

Climate forecasts are not available at high spatial resolution; therefore the epidemic warnings employing such forecasts will be at a relatively coarse geographical scale. Does the spatial scale of the information provided compromise the usefulness of seasonal forecasts in the region? The results indicate that this is the case for forecasts of rainfall, but not for forecasts of temperature. The August DEMETER rainfall forecast predicted only average rainfall for the 1997–1998 season over the grid point covering Kagera region. In contrast, there is much better agreement between forecast anomalies of temperature for the first season and malaria incidence. Over the nine-year period, the DEMETER temperature forecasts compared very well to the observed temperatures. These forecasts were able to predict high malaria incidence years during the first season despite the low statistical significance of the temperature relationship. For the second season, the rainfall forecasts were more closely associated with observations than for the first season, although DEMETER failed to predict the most extreme years. The long time lag between observed temperature anomalies and the corresponding correlated malaria anomalies meant that forecasts of temperature were not required for this season, however, DEMETER rainfall forecasts were able to enhance the performance beyond the use of observed temperature anomalies alone due to the capability of the DEMETER rainfall forecasts in predicting the intermediate (i.e. non-extreme) years.

## Conclusion

Seasonal forecasts have become more readily available to the users' community in recent years. Nevertheless, few studies have been undertaken to assess their potential benefits for the health sector, probably because of the lack or difficulty in obtaining epidemiological datasets of sufficient length and the multidisciplinary nature that such research involves. Using monthly malaria data collected from a district hospital, the linkage between climatic data and epidemics of malaria was investigated in the Kagera region of NW Tanzania. The ability of the DEMETER seasonal forecasting system in predicting the climatic conditions found to be correlated with malaria epidemics was then assessed. This analysis reveals the importance of seasonal temperature forecasts in malaria early warning systems and the apparent skill of those forecasts. This research also points towards the need for downscaled rainfall forecasts to become available, particularly in regions of known predictability such as East Africa.

## Competing interests

The author(s) declare that they have no competing interests.

## Authors' contributions

AEJ participated in the design of the study, extracted the seasonal forecasts from the DEMETER models, performed the statistical analyses, wrote a draft of the results section, and contributed to the editing of the final paper.

UUW collected the epidemiological and climatic data in Tanzania, wrote a draft of the introduction and the materials sections, and contributed to the editing of the paper.

APM is the supervisor of AEJ. He participated in the design of the study and provided a thorough review of the manuscript.

IMH is the supervisor of UUW. He commented on the design of the study and provided substantial revisions to the drafts of the paper, and verified the statistical analyses.

ASG participated in the conception and design of the study, he contributed to the development of the statistical models with AEJ, and put the paper together based on sections provided by AEJ and UUW.

All authors have read and approved the final manuscript.
